# Magnetic targeting of microbubbles against physiologically relevant flow conditions

**DOI:** 10.1098/rsfs.2015.0001

**Published:** 2015-10-06

**Authors:** Joshua Owen, Paul Rademeyer, Daniel Chung, Qian Cheng, David Holroyd, Constantin Coussios, Peter Friend, Quentin A. Pankhurst, Eleanor Stride

**Affiliations:** 1 Institute of Biomedical Engineering, Department of Engineering Science, University of Oxford, Old Road Campus Research Building, Oxford OX3 7DQ, UK; 2 Nuffield Department of Surgical Sciences, University of Oxford, John Radcliffe Hospital, Oxford OX3 9DU, UK; 3 Healthcare Biomagnetics Laboratory, University College London, 21 Albemarle Street, London W1S 4BS, UK; 4 Institute of Biomedical Engineering, University College London, Gower Street, London WC1E 6BT, UK

**Keywords:** microbubbles, magnetic targeting, ultrasound, drug delivery, contrast agent, imaging

## Abstract

The localization of microbubbles to a treatment site has been shown to be essential to their effectiveness in therapeutic applications such as targeted drug delivery and gene therapy. A variety of different strategies for achieving localization has been investigated, including biochemical targeting, acoustic radiation force, and the incorporation of superparamagnetic nanoparticles into microbubbles to enable their manipulation using an externally applied magnetic field. The third of these strategies has the advantage of concentrating microbubbles in a target region without exposing them to ultrasound, and can be used in conjunction with biochemical targeting to achieve greater specificity. Magnetic microbubbles have been shown to be effective for therapeutic delivery *in vitro* and *in vivo*. Whether this technique can be successfully applied in humans however remains an open question. The aim of this study was to determine the range of flow conditions under which targeting could be achieved. *In vitro* results indicate that magnetic microbubbles can be retained using clinically acceptable magnetic fields, for both the high shear rates (approx. 10^4^ s^−1^) found in human arterioles and capillaries, and the high flow rates (approx. 3.5 ml s^−1^) of human arteries. The potential for human *in vivo* microbubble retention was further demonstrated using a perfused porcine liver model.

## 1. Introduction

Advances in the development of new types of pharmaceutical product have resulted in rapidly growing demand for more effective delivery systems. New delivery methods for existing products are similarly being sought to mitigate the impact of patent expiration [1]. While systemic delivery of a drug, e.g. by intravenous or oral administration has significant advantages in terms of convenience and cost, it can lead to harmful side effects [2]. Moreover, conventional administration methods are simply not suitable for several classes of therapeutic compound. These include poorly soluble drugs and large molecules such as proteins, which often produce a negligible therapeutic effect when delivered orally or intravenously [3].

There are three criteria that any drug delivery system should fulfil to provide maximum therapeutic efficacy with minimal unwanted side effects: (i) that it prevents unwanted damage and degradation of the therapeutic material during circulation, (ii) that it ensures the majority of the material is maintained at the desired location(s), and (iii) that it promotes entry of the therapeutic compound into the target tissue [4].

There have been a large number of studies in recent years demonstrating the considerable potential of coated microbubbles as agents for drug delivery [5,6]. Long established as efficient contrast agents for ultrasound imaging [7], microbubbles have been widely shown to improve both extravasation and the cellular uptake of therapeutic material [8–10]. However, for microbubble-enhanced delivery to be effective, there must be a sufficient concentration of microbubbles at the target site. Simply increasing the systemic microbubble concentration is undesirable as it can increase the risk of embolism and shield target tissue from ultrasound exposure [11].

To address this challenge, various strategies for targeting microbubbles to specific sites have been explored. Microbubbles have been successfully targeted *in vitro* via electrostatic coupling [12], molecular binding through the use of antibodies and proteins [13], and acoustic radiation force [14]. However, efficient targeting of microbubbles still represents a considerable challenge *in vivo*, as the surface architectures that maximize targeting typically also increase the presentation of immunogenic compounds, which can lead to early particle clearance or a hypersensitivity response [5]. An alternative method of targeting which has shown considerable potential uses microbubbles with superparamagnetic nanoparticles incorporated into their coating.

The use of both micro- and nano-scale magnetic particles has been explored for the delivery of therapeutic agents for several decades [15–18], and more recently for gene delivery [19]. In 2000, Soetanto & Watarai [20,21] demonstrated electrostatic conjugation of stearate-coated magnetic microparticles to microbubbles stabilized with the same material via calcium ion binding. Magnetic microbubble formulations have since been developed for dual-purpose ultrasound and magnetic resonance imaging (MRI) contrast agents, and as drug delivery vehicles [22–25]. In 2009, Stride *et al*. [26] published a study in which magnetic microbubbles were used for gene delivery to Chinese hamster ovary cells. Magnetic microbubbles, non-magnetic microbubbles and/or magnetic liquid droplets were co-injected with naked plasmid DNA encoding for luciferase and the cells exposed to a magnetic field, ultrasound or both. It was found that the highest rates of transfection were achieved with simultaneous exposure to ultrasound and a magnetic field with magnetic microbubbles [26]. This formulation was also successfully used to deliver a bioluminescent marker to the right lung of a mouse *in vivo* [27]. Vlaskou *et al*. [24] similarly used magnetic and acoustically active lipospheres to deliver therapeutic agents *in vitro* and *in vivo* under the application of ultrasound.

Magnetic microbubbles have thus demonstrated considerable potential as delivery agents, but it is unknown yet whether they are capable of being targeted under the flow conditions typically found in the human body. Evidence of targeting in small animal models is of limited relevance, as successful targeting of magnetic particles requires the combination of magnetic field strength and gradient to be sufficiently high at the relevant tissue depth. In most cases this will be significantly greater in humans. Moreover, there is a rapid reduction in magnetic force with distance from the magnet.

The aim of this study was therefore to investigate targeting of magnetic microbubbles under flow conditions and length scales relevant to the human body. The ability of microbubbles to be retained was determined in vessels of different diameters and under flow conditions ranging from the high shear rates found in the capillaries to the high flow rates found in the arteries. The impact of substituting the suspending liquid for whole blood was then examined, and finally a preliminary experiment was performed in a perfused porcine liver.

## 2. Theoretical modelling

**Figure 1.**
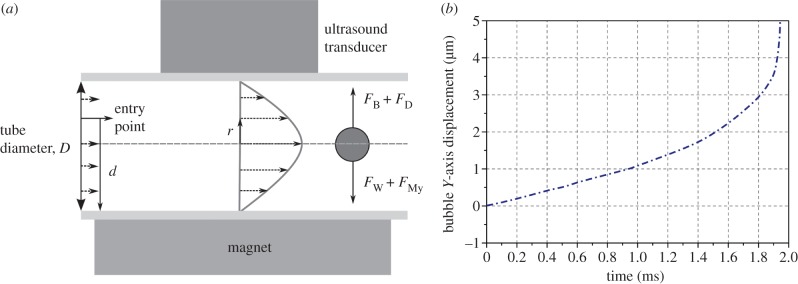
(*a*) Schematic of geometry used in the theoretical modelling and (*b*) example of microbubble trajectory generated by the numerical simulations.

Prior to commencing the experimental work, numerical simulations were performed to estimate the flow conditions under which magnetic targeting of microbubbles should be theoretically possible. From conservation of momentum, the vertical motion of a single spherical particle suspended in an incompressible, single-phase Newtonian liquid undergoing steady, laminar flow in a horizontal cylindrical vessel in the presence of a magnetic field with constant gradient (figure 1) may be written as
2.1
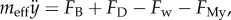

where *m*_eff_ is the effective mass of the particle and *F*_B_, *F*_D_*, F*_w_ and *F*_My_ refer to the vertical forces acting on it due to buoyancy, viscous drag, its weight and the magnetic field gradient, respectively.

If the particle is a bubble with a gas core of radius *R*_1_ surrounded by a liquid shell of thickness *R*_2_ − *R*_1_ that contains a volume fraction *α* of magnetic nanoparticles then equation (2.1) may be re written as2.2
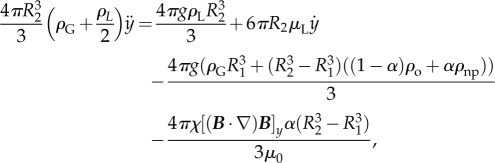

where *ρ*_G_ is the density of the gas, *ρ*_L_ and *μ*_L_ are the density and viscosity of the surrounding liquid, respectively, *ρ*_o_ is the density of the shell material, *ρ*_np_ is the density of the magnetic nanoparticles and *χ* is their effective volumetric susceptibility, ***B*** is the magnetic field, *g* is the acceleration due to gravity and *μ*_0_ is the permeability of free space.

Solving this system of equations enables the time required for the bubble to travel from its initial position in the vessel to the wall closest to the magnet to be determined. Provided this time *t*_r_ is shorter than the time taken for the bubble to flow out of the target region defined by the distance over which the magnet provides sufficient force, *L*_m_ then the bubble has the potential to be retained (i.e. it is necessary that 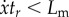). Simulations were performed using a fourth order Runge–Kutta solver in the Matlab^®^ numerical computing environment (2012B, The MathWorks, Natick, MA, USA). A range of variables describing different combinations of bubble size and magnetic nanoparticle content, liquid flow rate, vessel diameter and region, and magnitude of constant magnetic force was used (table 1). The parameters for the magnetic microbubbles were based on the formulation published in [26] (see §3.1) and the ranges of diameters and volume flow rates were selected to mimic conditions in different types of blood vessel (see §3.2).

**Table 1.** Summary of parameters used in the numerical simulations.

quantity	symbol	units	value(s)
density of the gas core (air)	*ρ*_G_	kg m^−3^	1.24
density of the coating liquid (isoparaffin)	*ρ*_o_	kg m^−3^	700
density of the nanoparticles (Fe_3_O_4_)	*ρ*_np_	kg m^−3^	5100
effective volumetric susceptibility of the nanoparticles	*χ*	units	0.85
volume fraction	*α*	—	0.1
density of the surrounding liquid (plasma)	*ρ*_L_	kg m^−3^	1025
viscosity of the surrounding liquid	*μ*_L_	Pa s	0.0015
acceleration due to gravity	*g*	m s^−2^	9.81
permeability of free space	*μ*_0_	T m A^−1^	1.26 × 10^−6^
magnetic field gradient product	[(***B***.∇)***B***]*_y_*	T^2^ m^−1^	18
gas core radius	*R*_1_	M	1–2 × 10^−6^
coating thickness	*R*_2_*–R*_1_	m	5 × 10^−8^
vessel inner diameter	*D*_v_	m	1–6 × 10^−3^
flow rate	*Q*_L_	m^3^ s^−1^	1–4 × 10^−6^
length of magnet	*L*_m_	m	0.05

It was assumed that the bubble would remain spherical and there would be no exchange of either gas or coating material with the surroundings. The bubble was treated as an incompressible particle and interactions with other bubbles and/or blood components were not considered. The validity of these assumptions is discussed later in the paper. The surrounding liquid was modelled as having the same properties as blood plasma. The motion of the particle was only considered in the vertical (*x, y*) plane parallel to the direction of flow (figure 1), and the flow velocity was assumed to follow a Poiseuille profile.

Once the bubble has reached the vessel wall, whether or not it can be retained there will depend upon the horizontal component of the magnetic force (not considered in the model), horizontal drag due to the flowing liquid and any adhesive or frictional forces between the bubble and the wall. These latter forces are, however, difficult to quantify accurately, and, therefore, the purpose of the simulations was only to determine the potential for retention. The results indicated that it should be possible to retain magnetic microbubbles of the size and composition described in [26] against a maximum flow rate of 3.3 × 10^−3^ m^3^ s^−1^ in vessels up to 6 mm in diameter with a magnetic field and gradient corresponding to the magnetic array used in [26] (i.e. for which [(***B***·**∇**)***B***]*_y_* = 18 T^2^ m^−1^).

## 3. Experimental material and methods

### 3.1. Preparation of magnetic microbubbles

1,2-Distearoyl-*sn*-glycero-3-phosphocholine (DSPC) was purchased from Avanti Polar Lipids Inc. (Alabaster, AL, USA). A ferrofluid suspension of 10 nm spherical magnetite nanoparticles in isoparaffin (10% volume fraction) was purchased from Liquids Research Ltd. (Bangor, UK). DSPC (15 mg) was weighed into a vial previously rinsed with surgical spirit (BP Unichem, Surrey, UK). Filtered deionized water (15 ml) was then added to the vial and the mixture sonicated using an ultrasonic cell disruptor (XL2000, probe diameter 3 mm; Misonix Inc., Farmingdale, NY, USA) at power setting 4 (15 s) followed by sonication at the air water interface (15 s). Fifteen microlitres of the 10 nm magnetite nanoparticle suspension was added followed by sonication (15 s in the liquid and 15 s at the air water interface) at power setting 4. The solution was then manually shaken for 30 s to produce magnetic microbubbles. As described in [26] these are hypothesized to consist of a gas core surrounded by the hydrophobic isoparaffin containing the magnetic nanoparticles and stabilized by and adsorbed layer of the amphiphilic phospholipid.

Samples of each type of microbubble were imaged under bright field optical microscopy to determine their size distribution and concentration. Ten microlitre samples were removed from three separate batches of each solution and examined on a haemocytometer (Bright-Line, Hausser Scientific, Horsham, PA, USA). Images were obtained with a 40× objective lens using a Leica DM500 optical microscope. The size distribution and concentration were then obtained using purpose written image analysis software in Matlab [28].

### 3.2. Flow models

As indicated above, successful targeting of magnetic microbubbles requires the magnetic force to be sufficient both to draw microbubbles to a target location and retain them there. Whether translation or retention of the microbubbles is the greater challenge will depend on the location of the target site. In larger vessels, it is likely to be the former, as both the average distance a bubble must travel to reach the wall and the flow rate will be higher. In the arterial system, for example, volume flow rates may be of the order of 10^−6^ m^3^ s^−1^ [29]. With decreasing vessel diameter, flow rate becomes less significant but the shear rate increases, being as high as 10^3^ s^−1^ in the capillaries and even higher in the arterioles [30]. The theoretical modelling indicated that magnetic microbubbles should have the potential to be retained at flow rates and vessel diameters up to those corresponding to medium-sized arteries. Confirmation of retention and in particular understanding of the effect of shear rate, however, needed to be obtained experimentally.

A series of different *in vitro* models was therefore used to simulate different flow conditions. To investigate microbubble targeting in larger vessels, latex tubes with inner diameters of 1.6, 3 and 6 mm were used. Latex was chosen because of its flexibility and because it is relatively transparent to ultrasound. For targeting in smaller vessels, optically transparent cellulose tubing (200 µm inner diameter) was used. These *in vitro* models clearly only mimic the most basic features of blood vessels, and there are numerous additional factors that could influence magnetic targeting. These include flow pulsatility, the rheological properties of blood and the mechanical and surface properties of the blood vessel wall. A preliminary examination of magnetic targeting in a more realistic *ex vivo* model was therefore also carried out using a perfused organ model. Each model is described in more detail in the following sections together with the different combinations of tubing diameters, volume flow rates and corresponding shear rates tested.

As above, it was assumed that flow in the vessel would be laminar, with a Poiseuille profile. The wall shear rate, *γ*, was therefore found as
3.1
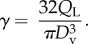



To determine the validity of this assumption, the Reynolds' number was also calculated for each set of flow conditions using
3.2
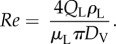



#### 3.2.1. Ultrasound flow phantoms

**Figure 2.**
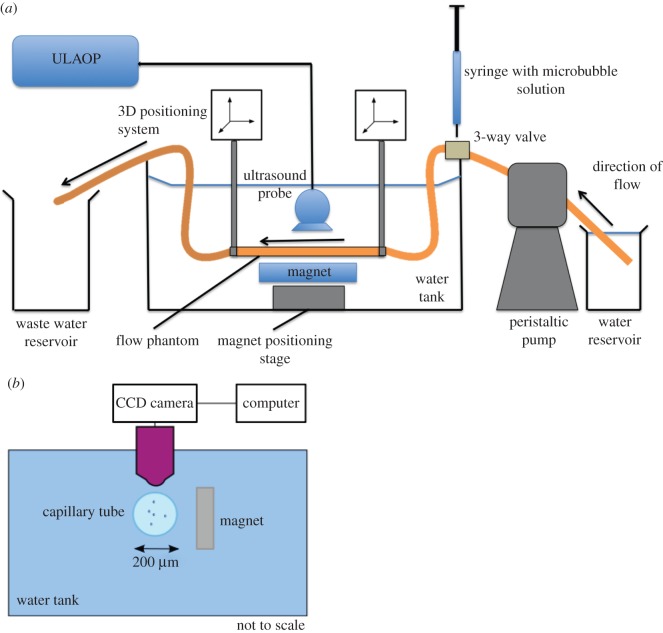
Schematic of flow phantom apparatus used in the experiments for (*a*) ultrasound imaging and (*b*) optical microscopy.

To investigate targeting in the larger flow phantoms, ultrasound imaging was used to observe the microbubbles. The apparatus was set up as shown in figure 2*a*. A latex tube (1.6, 3 or 6 mm inner diameter) was suspended in a water bath at the ambient temperature (23°C) and connected to either a peristaltic pump (Gilson MiniPuls3, Gilson, Luton, Beds. UK) drawing from a reservoir of the relevant suspending liquid, or a raised reservoir providing gravity fed flow for the highest flow rates. A section of the tube was positioned so that it was parallel to the base of the bath with a gap of approximately 3 cm to allow for the insertion of the magnetic array. A T-junction was inserted into the tubing to allow for the injection of magnetic microbubbles upstream of the magnet. The outlet of the tubing was fed to a waste reservoir at atmospheric pressure.

An ultrasound linear array transducer (9.4 MHz LA523, Esaote, Italy) was positioned above the section of tube under which the magnetic array was located in order to visualize the microbubbles. Video sequences were acquired using a ULA-OP ultrasound engine (Microelectronic Systems Design Laboratory, Universita degli Studi di Firenze, Florence, Italy) at a frame rate corresponding to a pulse repetition frequency of 8 kHz. The peak negative pressure at the focus was measured using a needle hydrophone (Precision Acoustics, 75 µm probe tip) as 0.3 MPa. Once a steady flow had been established in the tube, data were acquired for a few seconds to provide a baseline image, after which a 1.5 ml bolus of magnetic microbubbles was injected and data were acquired for a further 60 s. This process was repeated three times for each set of experimental conditions.

#### 3.2.2. Magnetic array

To provide the magnetic force, a Halbach array was used. The array comprised five N52 grade Nd_2_Fe_14_B permanent magnets (each 10 × 10 × 25 mm, supplied by NeoTexx, Berlin, Germany) with transversal magnetizations (1.5 T) at angles of 90° from one to the next, held in position in an aluminium frame. The performance of the as-made array was assessed against a finite-element model (using Opera-3D software, Cobham CTS Ltd, Oxford, UK) of the expected magnetic field and field gradient (see the electronic supplementary material). Reasonable agreement was found, with a measured field gradient at a point 2 mm above the centre of the array of 55 ± 5 T m^−1^, compared with a predicted gradient of 66 T m^−1^. The working field gradient 10 mm above the array was 32 T m^−1^.

#### 3.2.3. Capillary flow model

To investigate targeting at higher wall shear rates, an optically transparent cellulose tube (200 µm inner diameter, Cuprophan RC55 8/200, Membrana GmbH) was used. The flow velocity and hence wall shear rate in the capillary was controlled using a high-precision syringe pump. The tube was bonded to a blunt needle with cyanoacrylate adhesive and rinsed through with ethanol. This was then attached to a 100 µl World Precision Instruments glass syringe inserted into a Harvard Apparatus PHD 2000 Infuse/Withdraw syringe pump. The capillary tube was submerged in a water bath at the ambient temperature and observed via a 40 × water immersion objective (LUMPLFLN 40XW, Olympus Corp.) mounted on a microscope (World Precision Instruments, H602–240, Sarasota, FL, USA with a 10× eye piece) (figure 2*b*). The end of the tube was inserted into a reservoir filled with 450 µl of the relevant suspending liquid and containing 50 µl of the magnetic microbubble suspension. The syringe was set to withdraw and liquid was drawn through the tube at a constant rate that was varied between 1.7 and 8.4 µl s^−1^. A single N52 grade NdFeB permanent magnet was positioned 1 mm from the tube wall giving a field of 0.37 T and gradient 78.5 T m^−1^ at the wall. Video footage of the microbubbles was recorded using a digital camera mounted on the microscope eyepiece (DCU224M, ThorLabs Ltd). Again the experiment was repeated three times for each flow rate.

#### 3.2.4. Targeting in blood

The majority of the experiments were carried out in phosphate-buffered saline (PBS). In a previous study by the authors, however, it was shown that microbubble targeting may be substantially reduced in whole blood compared with PBS [31]. A subset of the experiments, corresponding to the higher flow and/or shear rates was therefore repeated with the microbubbles suspended in whole porcine blood. White Landrace pigs weighing 45–60 kg were used for blood donation and were treated in accordance with the United Kingdom Animals (Scientific Procedures) Act 1986. The internal jugular vein and carotid artery were cannulated following isofluorane induction of general anaesthesia and endotracheal intubation. Heparin (20 000 Units; CP Pharmaceuticals, UK) was administered intravenously, and a Gelofusine^TM^ (B Braun, UK) infusion was commenced via the central venous line. Autologous donor blood was collected via the aortic cannula and was stored in dextrose-supplemented citrate blood transfusion bags (CPDA-1 Single Blood Collection Systems; Fenwal, USA) at 4°C for subsequent use.

#### 3.2.5. Perfused liver targeting

An extracorporeal normothermic liver perfusion device, which was developed for organ preservation prior to transplantation and which can maintain a liver in a functional state for in excess of 72 h *ex vivo*, was used to provide a more physiologically relevant model [32]. A porcine liver was chosen as it is widely accepted to be the most representative preclinical model [33]. One advantageous feature of the liver perfusion device was that vascular flow rates could be controlled precisely, or perfusion could be stopped entirely by turning the device off and clamping the inflow/outflow tracts. Details of the retrieval process and perfusion system may be found in the electronic supplementary material. Following approximately 30 min of normothermic machine perfusion, the liver was placed in an acoustically transparent 50 × 50 cm sterile intestinal bag (3 M, USA) filled with isotonic colloid solution (Gelofusine, Braun, UK), which had been degassed and pre-heated to 37°C. The bag was suspended in a silicone sling over a water bath containing an acoustic absorber in the base, which was continuously degassed and heated to 37°C.

A suitable blood vessel was located using a linear array probe (model L10-5; Zonare Medical Systems, Mountain View, CA, USA) with 128 elements, 38 mm aperture and 5–10 MHz bandwidth attached to an ultrasound engine (model z.one; Zonare Medical Systems) and the magnetic Halbach array was inserted underneath the liver in as close proximity to the liver as possible, giving a magnetic field of 0.05 T at the vessel wall. From the theoretical modelling and *in vitro* experiments it was clear that this would be insufficient to retain microbubbles at the normal perfusion rate (approx. 6 ml s^−1^) and the flow rate in the vessel was therefore reduced (to approx. 0.3 ml s^−1^) by partially clamping the inflow tracts. The ultrasound probe was held in position with a clamp and the vessel was cannulated with a 22G hypodermic needle. A 1.5 ml bolus of the magnetic microbubble suspension was injected through the needle and data recorded as in the flow phantom experiments. The experiment was repeated in a second liver.

### 3.3. Image processing

#### 3.3.1. Ultrasound imaging

Previous studies have demonstrated that ultrasound image intensity is directly proportional to microbubble concentration for clinically relevant values (10^4^–10^5^ microbubbles ml^−1^) [34]. As far as possible, the size distribution, concentration and volume of the microbubble suspensions were kept constant throughout the study. Therefore, it was deemed reasonable to assume that image intensity would provide an indication of the quantity of microbubbles retained in these experiments. It is likely that the microbubble concentrations produced by magnetic targeting actually exceeded the limiting value for a linear relationship between image intensity and concentration [35]. This would have led to an underestimate in the number retained. As, however, it was the relative change in intensity that was of interest for each set of experimental conditions the resulting uncertainty was considered to be acceptable.

**Figure 3.**
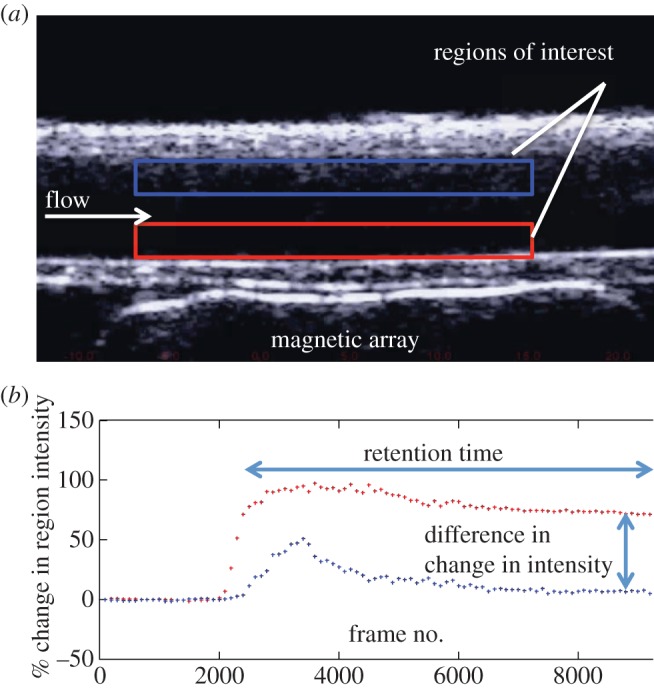
(*a*) Example of image obtained in ultrasound flow phantom showing regions of interest at the upper and lower surfaces of the tube and (*b*) example of graph showing change in intensity within both regions of interest (microbubbles arrive at the section of the tube under the ultrasound probe after approx. 2000 frames).

A purpose-written Matlab program was used to quantify the retention of the microbubbles by the magnet. Two rectangular regions of interest were defined at the lower and upper surfaces of the tube (figure 3*a*). If microbubbles are successfully retained, then the intensity of the former should increase over time while that of the latter should remain the same. The change in intensity over the course of each experiment was measured and plotted as shown in figure 3*b*. The average steady-state change in intensity was determined for each set of conditions. For drug delivery to occur, magnetic microbubbles must be retained both in sufficient concentration and for a sufficient length of time for therapeutic effects to be realized. Therefore, the time over which the increase in intensity was sustained was also determined.

#### 3.3.2. Optical imaging

The number of microbubbles retained in the capillary tube at each flow rate over a period of 4 min was determined, again using a purpose-written Matlab program. In practice (see §4.3), the number of magnetic microbubbles retained was such that individual bubbles could not be discerned. Therefore, the width of the retained microbubble bolus was measured from the images. This method of quantification will inevitably produce an underestimate of the number of targeted microbubbles as the image analysis was only performed in the focal plane. However, all measurements were relative and the focal plane was maintained between the experiments.

## 4. Results

### 4.1. Microbubble size distribution and concentration

**Figure 4.**
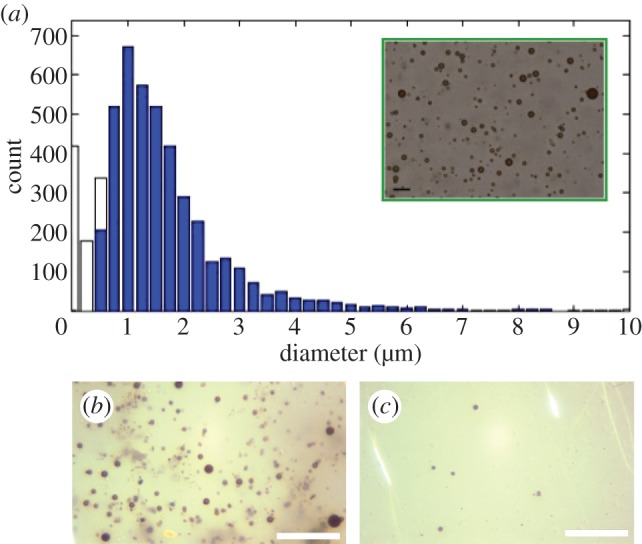
(*a*) Size distribution of magnetic microbubbles with insert showing an example of the images used to obtain it (unfilled bars indicate microbubbles detected whose size was smaller than the optical resolution of the system); (*b*) optical micrograph of magnetic microbubbles immediately before targeting and (*c*) after (the scale bar represents 40 µm in all images).

Figure 4*a* shows the average size distribution and concentration of the magnetic microbubbles obtained from the microscope images immediately following preparation. The modal diameter was between 1 and 2 µm and the mean concentration was approximately 10^7^ microbubbles ml^−1^. Measurements were performed before each set of experiments that confirmed consistency between the different batches.

It was important to determine whether or not magnetic targeting led to agglomeration of microbubbles, as the formation of large bubbles could potentially pose a risk of embolism. This was investigated by examining magnetic microbubbles before and after retention with the magnet in the capillary tube (figure 4*b*). Following removal of the magnet, the bolus of microbubbles was seen to disperse and the size distribution and appearance of the microbubbles were found to be indistinguishable from those before retention. No large bubbles were observed in any of the 30 images taken post retention.

### 4.2. Ultrasound imaging

In the results presented below, the intensity in each region of interest is expressed as a percentage of its initial value to minimize the effect of any differences in image background intensity. ‘Retention time’ refers to the time for which the change in image intensity at the lower tube surface (nearest the magnet) is elevated above that of the upper by at least 5% (figure 3). The magnitude of this difference was also recorded.

#### 4.2.1. Retention time

**Figure 5.**
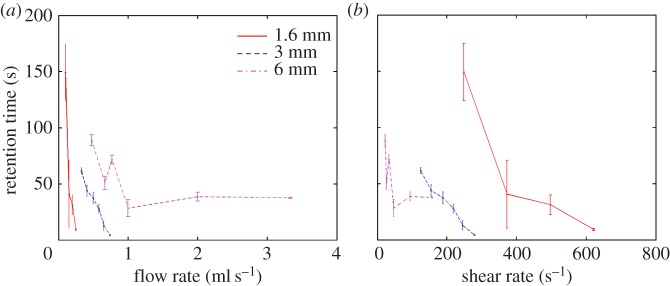
Variation in retention time with (*a*) volume flow rate and (*b*) shear rate for different tubing diameters.

Figure 5*a* shows the variation in retention time with flow rate for a magnetic field along the bottom of the tube of 0.2 T. In the 1.6 mm flow phantom, as the flow rate increases the retention time decreases linearly until at 0.25 ml s^−1^ the retention time is just 10 s. At higher flow rates negligible retention of microbubbles was observed. Similar results were observed in the 3 mm phantom, with retention time reducing from 1 min at 0.3 ml s^−1^ to approximately 5 s at 0.75 ml s^−1^. In the 6 mm phantom, the relationship between retention time and flow rate is more complicated with retention time decreasing irregularly and then maintaining a value of approximately 40 s above 1 ml s^−1^ up to 3.34 ml s^−1^. It was not possible to generate a higher flow rate with the apparatus available.

#### 4.2.2. Difference in intensity change

**Figure 6.**
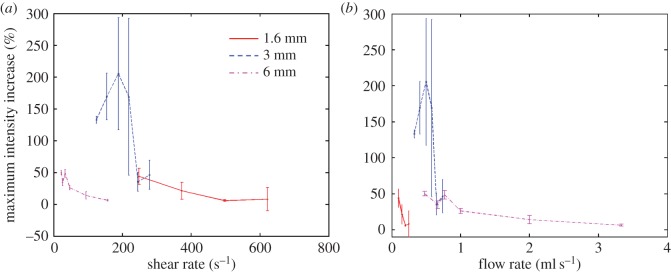
Variation in difference in intensity change with (*a*) volume flow rate and (*b*) shear rate for different tubing diameters.

Figure 6*a* shows how flow rate affected the difference in the change in image intensity produced by magnetic targeting with a magnetic field of 0.2 T and field gradient of 32 T m^−1^ between the upper and the lower tube surfaces. Again an initially linear relationship was seen in the 1.6 mm flow phantom with a difference in intensity change of approximately 45% at 0.1 ml s^−1^ falling to 10% at 0.34 ml s^−1^. An overall reduction in the difference in intensity change with flow rate was also seen in both the 3 and 6 mm phantoms, but there was much greater variability in the results. The data shown in both figures 5*b* and 6*b* suggest that tube diameter determines whether shear rate or flow rate has the strongest effect upon targeting. The results indicate that shear rate is the limiting factor in the 1.6 mm flow phantom, a combination of shear rate and flow rate limits targeting in the 3 mm phantom and flow rate is the dominant factor in the 6 mm phantom.

#### 4.2.3. The effect of blood

As expected from the authors' previous work [31], blood was found to significantly affect the retention of magnetic microbubbles. Table 2 shows the difference in the change in image intensity in the 1.6 mm flow phantom at a flow rate of 0.1 ml s^−1^ in water and whole blood. In the case of the former, a difference of more than 40% in the intensity change was seen. This was reduced to 18% in whole blood. Similarly the retention time was reduced from 149 s to 101 s. The authors have previously hypothesized that this is due to collisions between microbubbles and red blood cells both inhibiting the translation of microbubbles towards the magnet and limiting retention. Similar results were seen in the 3 and 6 mm phantoms and in each of three repeats for the sets of conditions tested.

**Table 2.** Comparison of targeting time and difference in intensity change produced by magnetic targeting of microbubbles in water and whole porcine blood.

diameter (mm)	liquid	mean difference in intensity change (%)	standard deviation (%)	retention time (s)	standard deviation (s)	flow rate (ml s^−1^)	shear rate (s^−1^)
1.60	water	44	13	149	26	0.10	249
1.60	blood	18	3	101	17	0.10	249
1.60	water	21	13	41	30	0.15	373
1.60	blood	11	1	7	1	0.15	373
1.60	water	6	2	31	9	0.20	497
1.60	blood	0	4	4	2	0.20	497
1.60	water	8	18	9	3	0.25	622
1.60	blood	NA	NA	NA	NA	0.25	622

### 4.3. Optical imaging

**Figure 7.**
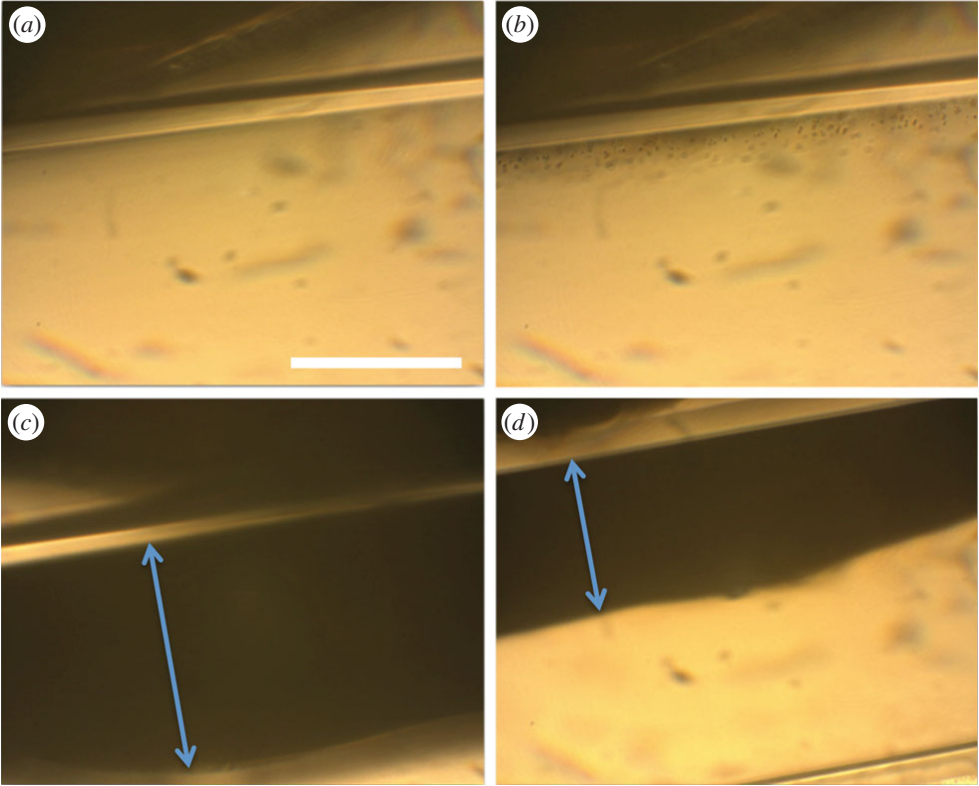
Optical micrographs showing retention of magnetic microbubbles in a 200 µm inner diameter cellulose tubing adjacent to a permanent magnet providing a magnetic field of 0.37 T and gradient 78.5 T m^−1^ at the tubing wall. (*a*) Before injection of microbubbles, (*b*) immediately following injection of microbubbles at a shear rate of approximately 2100 s^−1^, (*c*) 30 s after injection at a shear rate of approximately 2100 s^−1^ and (*d*) 30 s after injection at a shear rate of approximately 11 000 s^−1^ (scale bar indicates 100 µm). The double headed arrows indicate the width of the microbubble bolus formed.

As discussed above, the influence of shear rate appears to become increasingly important with reducing vessel diameter. It was therefore important to determine the potential for magnetic targeting in a smaller flow phantom at higher shear rates. Ultrasound imaging was not possible in tubing significantly smaller than 1.6 mm. Magnetic targeting was therefore examined in the 200 µm tubing using an optical microscope. The magnet was placed directly alongside the tubing and the centre of the tubing next to the magnet located in the focus of the microscope. Single frames from the video footage captured of microbubbles being retained in the 200 µm cellulose tubing are shown in figure 7. At a flow rate and shear rate of 1.7 µl s^−1^ and approximately 2000 s^−1^ respectively a large bolus of magnetic microbubbles was formed over approximately 3 min which extended 150 µm into the tube lumen. Even at the highest flow rate of 8.4 µl s^−1^ corresponding to a shear rate of over 11 000 s^−1^ retention was still observed, with a bolus of 65 µm being formed. In contrast to the ultrasound experiments, there was no reduction in the bolus width observed, even several minutes after its formation. Substituting whole blood as the suspending liquid led to a decrease in the size of the retained bolus as observed in the previous study [31].

### 4.4. Targeting in a perfused liver

**Figure 8.**
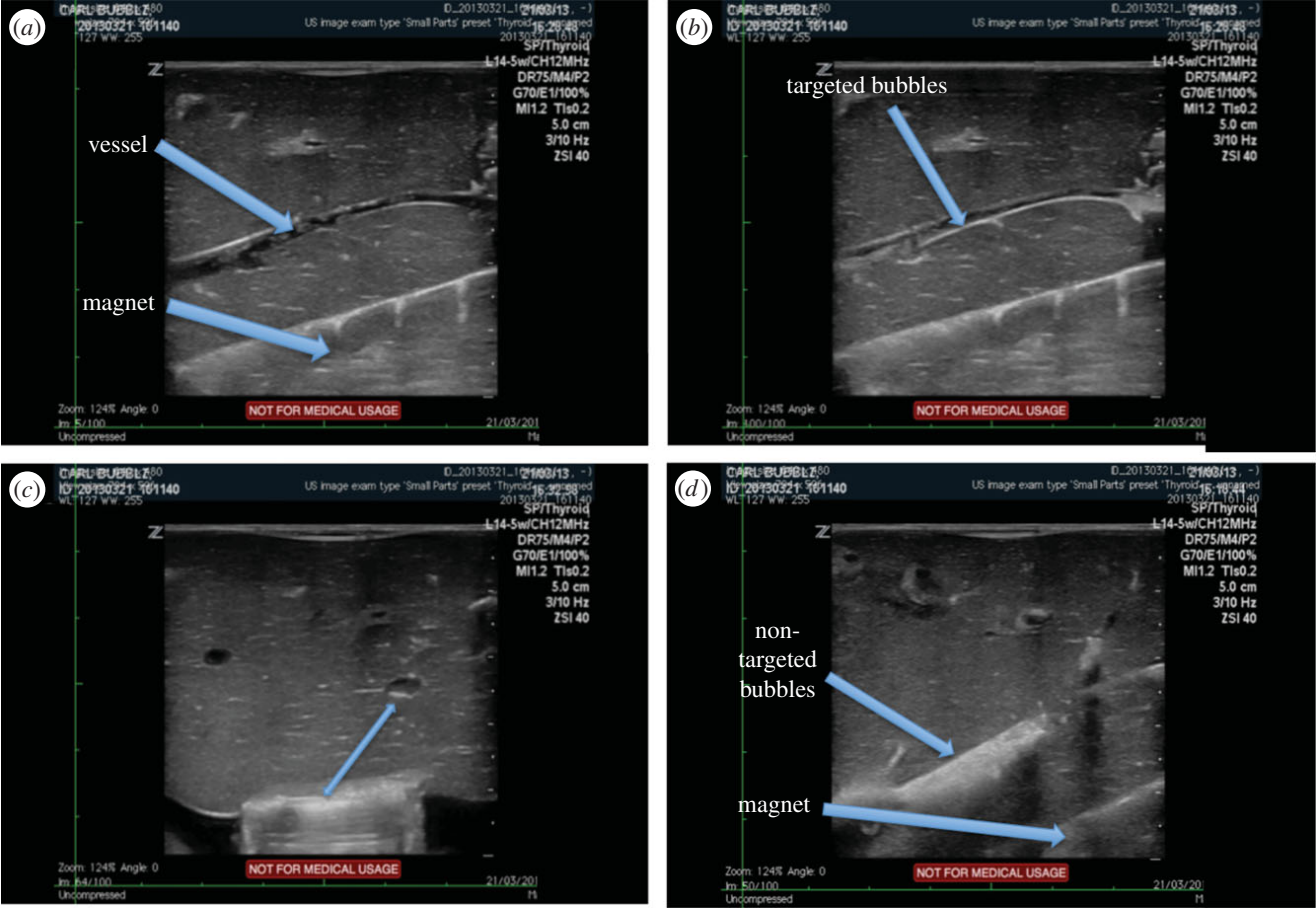
Ultrasound images showing a blood vessel in an *ex vivo* perfused liver model with a magnetic Halbach array positioned underneath it. (*a*) Before injection of magnetic microbubbles, (*b*) showing magnetic microbubbles retained at the vessel wall, (*c*) transverse view showing misalignment of the magnetic array and (*d*) non-magnetic microbubbles flowing through the vessel (second liver).

The purpose of this experiment was to determine whether magnetic microbubbles could still be retained in a more physiologically relevant model. In particular it was important to confirm whether or not extravasation/translation into the microvasculature would occur. While extravasation may be desirable for therapeutic delivery, as discussed in the introduction, it is important to be able to control the process spatially and temporally. In this respect, ultrasound-mediated extravasation would be preferable to that promoted by a static magnetic field on account of the ability to focus the ultrasound field and the timescales associated with cavitation [36]. In both livers, microbubbles were seen to be retained at the lower (magnet-side) wall of the vessel in a similar manner to that observed in the flow phantom (figure 8). Unfortunately, the Halbach array could not be inserted and removed while maintaining the same field of view. Therefore, non-magnetic microbubbles (SonoVue^®^) had to be used for comparison, but no retention was observed and as expected (owing to the inherent buoyancy of the microbubbles), the maximum intensity was seen at the upper surface of the vessel. There was no evidence of extravasation, nor was there evidence of microbubbles being drawn into the microvasculature with either type of bubble.

### 4.5. Summary of targeting limits

The maximum flow rates and shear rates against which magnetic microbubbles could be retained are shown in table 3 with the corresponding magnetic field parameters. The minimum criteria for successful retention were that, first, the intensity at the lower surface should increase over that at the upper surface and, second, that a difference in the change in intensity of at least 5% should be sustained for more than 1000 frames.

**Table 3.** Summary of maximum volume flow rate and shear rate at which magnetic targeting of microbubbles was observed.

tube diameter (mm)	fluid	max flow rate (ml s^−1^)	shear rate (s^−1^)	magnetic field strength (T)
6.0	water	3.34	157	0.20
3.0	water	0.74	272	0.20
1.6	water	0.20	622	0.20
0.2	water	0.0084	10 700	0.37

The results show that magnetic microbubbles could be retained at a flow rate of 3.34 ml s^−1^ in the 6 mm tube with a magnetic field of 0.2 T at the lower surface of the tube and a gradient of 32 T m^−1^. This corresponds to conditions found in small to medium-sized human arteries [29]. The maximum shear rate at which magnetic targeting was observed was in the 200 µm tubing. This is of the same order of magnitude as that seen in the arterioles.

As this had already been investigated in a previous study [31], the flow rate and shear rate at which magnetic microbubbles could be retained in whole blood were only determined in the 1.6 mm flow phantom. The maximum shear rate against which targeting could be detected (i.e. for which there was a difference of more than 5%) decreased by over 100 s^−1^ and the maximum flow rate was reduced from 0.2 to 0.15 ml s^−1^.

## 5. Discussion

Both the theoretical modelling and *in vitro* results indicate that magnetic targeting of microbubbles can be successfully achieved under flow conditions corresponding to a significant proportion of blood vessels in the human body. The combinations of vessel diameters, flow rates and shear rates at which targeting was observed encompass all veins and venules, the larger capillaries and smaller arteries.

To the best of the authors' knowledge only one other study has examined the targeting of magnetic microbubbles *in vitro* [24], and that was under less challenging conditions. As described above, the study used magnetic acoustically active liposomes (MAALs) in saline in a 1 mm inner diameter tube with a flow rates between 0.01 and 0.15 ml s^−1^. An 0.9 T electromagnet with a field gradient of 100 T m^−1^ was placed directly below the tube and magnetic targeting was detected by examining the volume fraction of bubbles before and after passing through the tube. There was no direct observation of retention. The study concluded that MAALs could be retained against a flow rate of 0.15 ml s^−1^. This is equivalent to a shear rate of approximately 191 s^−1^.

The relevance of the results obtained to the clinical applicability of magnetic targeting clearly depends on the desired application and/or therapeutic target. Microbubbles have been proposed as agents for sonothrombolysis (both as drug carriers and for cavitation nucleation) [37], delivery of large molecules (DNA, siRNA) and other agents (e.g. oncolytic viruses) to a variety of targets [36,38], and delivery and/or enhanced penetration of chemotherapy [5]. For many of these applications, the vessel diameters and corresponding shear rates and flow rates fall into the range investigated in this study. For delivery within some tumours, however, targeting in capillaries with diameters smaller than those examined will be required. The maximum shear rates and flow rates at which microbubbles could be retained (table 3) are in fact higher than those found in smaller capillaries; but other factors may become significant with decreasing vessel size. These may include, for example, the mechanical properties of the vessel wall and also interaction with blood cells. As demonstrated in both this study and the authors' previous study [31], blood reduces the quantity of microbubbles retained for a given set of flow conditions and magnetic field parameters. The most likely explanation is collisions between microbubbles and blood components impeding the translation of the former. This effect would be expected to become increasingly significant as the vessel size becomes comparable with that of an erythrocyte (approx. 8 µm). Unfortunately, only one type of tubing was available with the required combination of flexibility and optical transparency for this study, but it is a matter that clearly requires further investigation.

Another subject that requires further attention is the relatively high variability indicated in figure 6, i.e. the relative change in image intensity. The microbubble fabrication protocol enabled the size distribution and concentration of the microbubbles to be kept consistent throughout the experiments. The quantity of magnetic nanoparticles encapsulated within each bubble, however, was much more difficult to control; and this would have affected both their ability to be retained by a magnetic field and their acoustic response [39]. It was clear when observing a population of magnetic microbubbles exposed to a magnetic field under an optical microscope that there was considerable variation in response (data not shown). It should be possible to address this limitation by modifying the fabrication method to ensure more uniform incorporation of the magnetic nanoparticles into the microbubble coating. This is currently under investigation by the authors. Alternatively, methods such as microfluidic processing [40] could be used to improve microbubble uniformity although these methods currently suffer from low production rates and processing particulate material can be challenging due to the high probability of clogging.

A further source of variability, again particularly in the ultrasound imaging results, was the dependence of the image processing on the selection of the region of interest. The significant difference in retention time obtained from the ultrasound and optical imaging also suggests that some microbubble destruction was occurring as a result of ultrasound exposure; although this could also have been related to differences in the horizontal component of the magnetic force which may have been higher in the smaller vessel. A further useful inference from comparing the retention of microbubbles using both ultrasound and optical imaging was that the role of acoustic radiation forces was negligible for the exposure conditions in this study. This was confirmed by increasing both the peak negative pressure (0.3–1.5 MPa) and pulse repetition frequency (0.5–8 kHz) (see the electronic supplementary material). In the case of the latter, there was no correlation with either retention time or difference in intensity change. In the case of the former, both quantities were reduced rather than increased as would have been expected had either the primary radiation force been assisting bubble translation and/or had the secondary radiation forces been promoting accumulation. The most likely reason is that there was increased microbubble destruction.

The maximum field values used in the experiments (0.2 T for ultrasound imaging, 0.37 T for optical imaging and 0.05 T for the perfused liver) were actually very low compared with the static fields typically found in MRI machines (1.5 T in hospitals and up to 9 T in research systems). The field gradients were relatively high but smaller than those used in other magnetic targeting studies (e.g. [24]). This indicates that it should be possible to compensate for the reduction in retention in the presence of blood by increasing the field and/or gradient. This is in contrast to biochemical targeting where it is not possible to increase the number of binding sites. Similarly, targeting should be feasible at higher flow rates and/or greater tissue depths. The latter is further supported by studies of other magnetically responsive microparticles [17]. In terms of safety, the magnetic nanoparticles used are similar to those currently used as MRI contrast agents and whose toxicity profile and clearance mechanisms are relatively well understood. The potential for additional risk due to magnetic actuation has also been examined previously and the existing evidence would indicate this is very small [41].

The experiments in the perfused liver highlighted the importance of correctly aligning the magnetic array with respect to the target vessel. Rotation of the ultrasound probe demonstrated that the orientation of the array was such that the magnetic force was not maximized at the wall of the vessel. This could be addressed by substitution of the basic Halbach array for a three-dimensional (3D) configuration and integration between the array and the ultrasound probe. Although there was no evidence of extravasation, the use of multiple magnets to achieve 3D manipulation of the microbubbles would be advantageous in avoiding unwanted transport of the bubbles, e.g. from large vessels into the microvasculature.

## 6. Conclusion

The results of this study indicate that magnetic targeting of microbubbles can be achieved under flow conditions relevant to human physiology using magnetic fields that are safe for clinical use. With a maximum field of 0.2 T and gradient of 32 T m^−1^, microbubbles were successfully retained in vessel phantoms with diameters ranging from 200 µm to 6 mm and at combinations of shear rates and flow rates corresponding to the larger capillaries, all veins and venules and small arteries. When the microbubbles were suspended in whole blood rather than saline, the quantity of microbubbles retained at a given combination of vessel diameter, flow rate and shear rate was reduced. Larger magnetic fields and field gradients and/or more magnetically responsive microbubble formulations will therefore be required to compensate for this effect; but this should not present a significant challenge given the field parameters used in existing MRI systems. Retention of microbubbles was also demonstrated in an *ex vivo* perfused porcine liver model, with the additional beneficial features that there was no evidence of any microbubble extravasation out of the blood vessels, nor was any agglomeration observed—both of which are important safety considerations when it comes to the translation of this technology into clinical use.

## Data accessibility

All data and methods are reported within this paper, the electronic supplementary material and at doi:10.5072/bodleian:xp68kj24v.
